# Sex-specific responses to sexual familiarity, and the role of olfaction in *Drosophila*

**DOI:** 10.1098/rspb.2013.1691

**Published:** 2013-11-22

**Authors:** Cedric K. W. Tan, Hanne Løvlie, Elisabeth Greenway, Stephen F. Goodwin, Tommaso Pizzari, Stuart Wigby

**Affiliations:** 1 Edward Grey Institute, Department of Zoology, University of Oxford, South Parks Road, Oxford OX1 3PS, UK; 2 Department of Physics, Chemistry and Biology, Zoology, Linköping University, 58183 Linköping, Sweden; 3 Department of Physiology Anatomy and Genetics, University of Oxford, Parks Road, Oxford OX1 3PT, UK

**Keywords:** Coolidge effect, individual recognition, genetic relatedness, *Orco*, *Drosophila melanogaster*

## Abstract

Studies of mating preferences have largely neglected the potential effects of individuals encountering their previous mates (‘directly sexually familiar’), or new mates that share similarities to previous mates, e.g. from the same family and/or environment (‘phenotypically sexually familiar’). Here, we show that male and female *Drosophila melanogaster* respond to the direct and phenotypic sexual familiarity of potential mates in fundamentally different ways. We exposed a single focal male or female to two potential partners. In the first experiment, one potential partner was novel (not previously encountered) and one was directly familiar (their previous mate); in the second experiment, one potential partner was novel (unrelated, and from a different environment from the previous mate) and one was phenotypically familiar (from the same family and rearing environment as the previous mate). We found that males preferentially courted novel females over directly or phenotypically familiar females. By contrast, females displayed a weak preference for directly and phenotypically familiar males over novel males. Sex-specific responses to the familiarity of potential mates were significantly weaker or absent in *Orco*^1^ mutants, which lack a co-receptor essential for olfaction, indicating a role for olfactory cues in mate choice over novelty. Collectively, our results show that direct and phenotypic sexual familiarity is detected through olfactory cues and play an important role in sex-specific sexual behaviour.

## 1. Introduction

Because natural populations often exhibit a degree of viscosity, individuals can encounter their previous mates (i.e. ‘directly’ sexually familiar), and also novel members of the opposite sex that are from the same family and local environment as previous mates (i.e. ‘phenotypically’ sexually familiar). Recent work indicates that direct familiarity/novelty can play an important role in mating preferences in a diverse range of species. In males, a preference for sexually novel mates, a behaviour known as the Coolidge effect often associated with polygamous mating systems [1,2], has intuitive adaptive significance as sexual selection often favours males who mate with multiple females [3,4]. Females, on the other hand, might face a more delicate balance between the potential benefits, such as the increased genetic diversity of the offspring or bet-hedging effects [5], and the potential costs, such as mounting immune responses against the inseminations of different males [6], of mating with sexually novel males.

Mating preferences for or against phenotypically novel or familiar mates are likely to have similarly important, sex-specific consequences. For example, if recognition of closely related mates is subject to error, then preference for phenotypically novel mates (i.e. individuals that are unrelated and belong to a different local environment from the previous mates) could enable individuals to reduce the risk of mating repeatedly with the same mate. Males generally gain more reproductive success from successive matings with different females than from mating repeatedly with the same female (as in the Coolidge effect). In addition, a preference for phenotypically novel mates would probably increase offspring genetic diversity because phenotypically novel mates are less likely to be related to previous mates. Therefore in principle, phenotypic familiarity may have profound ramifications for mating preferences, the intensity of local mate competition, conflict and cooperation within and between the sexes, and ultimately the genetic structure of populations [7–10]. However, very little is known about the independent roles that direct and phenotypic familiarity may play in the mating preferences of males and females.

Here, we experimentally test male and female responses to both direct and phenotypic familiarity of mates in the fruit fly *Drosophila melanogaster*. Individuals of this species mate multiply [11] and natural populations are characterized by limited dispersal and a tendency towards aggregations in particular localities [12,13]. These factors are likely to increase the probability of an individual encountering their previous mates, as well as potential mates belonging to the same family and local environment as the previous mates. The use of *D. melanogaster* also provides us with the opportunity to use genetic tools to explore the proximate mechanisms underpinning differential responses to direct and phenotypic familiarity. A key candidate mechanism is olfaction, a sensory system that has a well-known role in species recognition in this taxon [14,15]. We address three aims: (i) to characterize male and female behavioural responses to direct familiarity; (ii) to establish male and female behavioural responses to phenotypic familiarity; and (iii) to test whether the gene *Orco*, which encodes a co-receptor essential for olfaction, is required for the behavioural responses to phenotypic familiarity.

## 2. Material and methods

### (a) Experimental population and culturing

We used a laboratory-adapted, wild-type, Dahomey stock of *D. melanogaster*, maintained since 1970 in large, outbred populations [16]. A *white*^Dahomey^ stock (in which flies possess white eyes) was derived by repeated backcrossing *w*^1118^ into the wild-type (red-eyed) Dahomey background [17]. The *Orco*^1^ loss-of-function allele [14,18] was backcrossed into the *white*^Dahomey^ genetic background for at least five generations to match the genetic background of the wild-type stock. Prior to experiments, the *white*^Dahomey^; *Orco*^1^ stock was backcrossed into the Dahomey stock to replace the *w*^1118^-bearing X-chromosome with the wild-type Dahomey X-chromosome to create experimental *Orco*^1^ flies. Thus, all *Orco*^1^ experimental flies possessed the wild-type (red) eye phenotype. Controls (also possessing the wild-type, red-eye phenotype) were *Orco*^+^ flies derived from the final generation of backcrossing. All flies were maintained in a 25°C, non-humidified room, with a 12 L : 12 D cycle, in plastic vials or bottles containing standard sugar–yeast medium with excess live yeast. Virgin flies were collected within 8 h of eclosion using ice anaesthesia. Larvae were raised at standard density (approx. 100 flies per bottle) [19].

### (b) Direct novelty experiments

Virgin adults were placed in same-sex vials for 5 days before conducting the ‘direct familiarity’ experiment. We tested whether males bias courtship toward females based on their direct familiarity or novelty by first mating males with a randomly chosen unrelated female and subsequently presenting each male with the female with which he had previously mated (the ‘directly familiar’ female) and a novel female that had previously mated with a different male (the ‘directly novel’ female; see the electronic supplementary material, figure S1AI). The initial matings were performed immediately after lights-on. The timings of the first matings of the two females were tightly matched, such that experimental females finished copulating within 15 min of each other, to avoid any potential biases that could be influenced by the time since mating (e.g. differences in female receptivity or pheromonal profile). Both females were randomly chosen from a large population so they were unlikely to be related to the male or to each other.

Three separate experiments were conducted to investigate the effect of direct familiarity on male behaviour. In the first and second experiments, we exposed the focal male to two live females (one novel and one familiar) in a plastic vial (93 mm high by 23 mm wide) containing standard sugar–yeast medium with excess live yeast immediately after the end of the first mating. Mating parameters (courtship counts, mating latency, mating duration and mating success) were recorded. Novel and familiar females were labelled by different methods, in a randomized balanced design. In the first experiment, females were either *white*^Dahomey^ (white-eyed) or wild-type (red-eyed) (*n*_males_ = 58); in the second, females were either marked with acrylic paint on their thorax or left unpainted (*n*_males_ = 88) [20]. In the third experiment, the focal male (*n*_males_ = 28) was presented with a choice of two mates (novel or familiar), which were decapitated and pinned via the thorax at 1 cm apart in the mating chamber (2 cm diameter and 1 cm height), within 30 min of the first mating. A ball of live yeast and a strip of filter paper soaked in distilled water were placed in the mating chamber to provide food and water for the male. This method enabled us to conduct detailed observation of male courtship of dead females [21], controlling for potential female influences on male courtship. Decapitated females do not extrude their ovipositor, depress, decamp or twist away from the male, thus they display a significantly reduced rejection response [21]. We recorded the occurrence of courtship events (chasing, singing, genital licking and copulation attempt) [22] directed at either female type (familiar or novel) in 15 min spot-checks until lights-off (12 h after lights-on) for the first and second experiments, or until a remating occurred. The third experiment was conducted blind with respect to the female type, and we quantified courtship counts at 1 min spot-checks for a duration of 4 h. We analysed variation in male courtship effort through generalized linear model (LM) with binomial error distribution. We randomly selected a focal female to avoid pseudoreplication and non-independence of data points, and we analysed the response variable ‘proportion of total courtship directed towards the female’. Each male was therefore represented only once in the dataset. ‘Female type’ (novel or familiar), ‘marked status’ (white-eyed or red-eyed; painted or unpainted) and its interaction with ‘female type’ were entered as fixed factors. We used R v. 2.13.0 for these analyses.

To examine female response to direct familiarity of males, we placed individual females in a mating chamber with two live males; a previous male mate (familiar) and a male that had previously mated to a different female (*n*_females_ = 43; see the electronic supplementary material, figure S1AII). We recorded female rejection behaviour towards the courtship of each male type [23] for 4 h. We analysed variation in female rejection behaviour using a generalized linear mixed model (GLMM) with binomial error distribution with ‘proportion of courtship rejected’ as the response variable, ‘male type’ (novel or familiar), ‘marked status’ and its interaction with ‘male type’ as fixed factors and ‘female identity’ as a random factor. We omitted data points in which both males were simultaneously courting the female because it was ambiguous as to which male the female was responding to. To test whether there was a difference in courtship intensity between the two male types, we used a GLMM with Poisson error distribution, ‘number of courtship events’ as the response variable, male type (novel or familiar) as a fixed factor and female identity as a random factor. R v. 2.13.0 was used for these analyses.

### (c) Phenotypic novelty experiments

Parental virgin males and females were paired in individual vials to produce families. The parental pair was discarded after 24 h and the eggs left to develop. The families were therefore only maintained for one generation (i.e. there was no inbreeding) and the offspring emerging from these vials were full siblings of one another and belonged to the same local rearing environment (i.e. the same vial). They were used for the ‘phenotypic familiarity’ and ‘olfaction’ experiments. Flies were approximately 3 days post-eclosion at the time of the first mating.

We first examined whether male flies discriminate between potential mates that are either phenotypically similar or phenotypically different to their previous mates. Focal males were first mated with females unrelated to themselves and thereafter presented with a choice of two virgin females: a female belonging to the same family and rearing environment as the first female (‘phenotypically familiar’) and a female belonging to a different family and local environment from the first mate (‘phenotypically novel’; see the electronic supplementary material, figure S1BI). In the first experiment, the focal male was placed in a vial with two live females (one phenotypically familiar and one phenotypically novel) which were marked on their thorax with either red or yellow acrylic paint [20] in a randomized balanced design (*n*_males_ = 36). We also conducted a second experiment (*n*_males_ = 79), in which females were decapitated and pinned in a mating chamber (see above). We recorded the number of courtship events directed at either female type in 1 min spot-checks until remating occurred in the first experiment, or for 4 h in the second experiment. In addition, for the first experiment, we recorded the remating latency and remating duration with either female type, together with the female type that eventually mated with the male. All trials were conducted blind with respect to female type.

Variation in courtship effort was analysed in the same manner as that of the ‘direct familiarity’ experiments. ‘Remating latency’ with either mate type was analysed using a Cox proportional hazards model with ‘latency to remating’ (time in minutes before second mating) as the dependent variable, ‘mate type’, ‘marked status’ and its interaction with ‘male type’ as fixed factors and ‘courtship proportion to mated female’ (number of courtship events directed to mated female divided by total number of courtship events by that male) as a covariate. The difference in remating duration with either mate type was tested with a general LM. ‘Mating success’ was analysed with *χ*^2^ tests for equal number of matings with either ‘mate type’ or ‘marked status’. The interaction of ‘mate type’ and ‘marked status’ on ‘mating success’ was assessed using a 2 × 2 *χ*^2^ contingency analysis. Where the number of matings was low, i.e. expected value less than 10, we used a Fisher's exact test. These analyses were conducted using JMP 9.0.

To investigate female response to phenotypic familiarity of males, each focal female (*n*_females_ = 40) was first mated to a male from a randomly chosen family unrelated to her, and subsequently placed with two novel males: a male belonging to the same family and local rearing environment as the female's first mate (‘phenotypically familiar’) and a male unrelated to the female and belonging to a different family and local rearing environment from the first mate (‘phenotypically novel’, see the electronic supplementary material, figure S1BII). Males were marked with paint, as described above. As a measure of female response, we recorded the latency to remating with either male type (phenotypically familiar or phenotypically novel) [24]. We also recorded the duration of remating and the type of male that mated with the female. Focal individuals that did not remate on the first day were separated from the two potential mates before lights-off and replaced into the experimental vials at lights-on the following morning. This procedure was repeated on subsequent days and the trial was concluded when at least 95% of the females remated. We tested the effect of relatedness on female response in this experiment using a Cox proportional hazards model [25] in R, with ‘latency to remating’ as the dependent variable and ‘male type’ (phenotypically novel and phenotypically familiar) as a fixed factor. ‘Marked status’ and its interaction with ‘male type’ were also entered as fixed factors in the model. Because female's latency to remating is likely to be influenced by the duration of the first mating [26] and the courtship intensity of the males [27], we entered the duration of first mating and courtship proportion by mated male as covariates. The survival curves were compared using the likelihood ratio test. We used a general LM to test the difference in remating duration with either mate type. ‘Mating success’ was analysed with *χ*^2^ tests for equal number of matings with either ‘mate type’ or ‘marked status’. Also, the interaction of ‘mate type’ and ‘marked status’ on ‘mating success’ was tested using a 2 × 2 *χ*^2^ contingency analysis.

In a second experiment to record female rejection behaviour, we placed mated females with two males (phenotypically familiar and phenotypically novel) in a mating chamber and recorded female response to the courtship attempts of each male type for 4 h (*n*_females_ = 59). Female rejection response was analysed in the same manner as that in the ‘direct familiarity’ experiment.

### (d) Olfaction experiments

**Figure 1.**
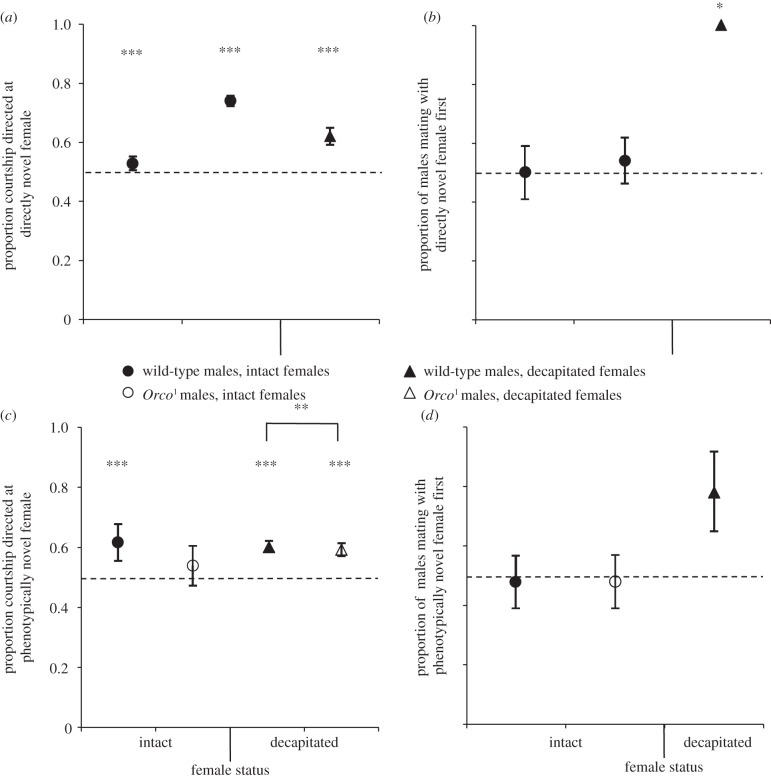
Male responses to direct familiarity and phenotypic familiarity of females. Dotted line drawn at proportion courtship = 0.5. Error bars denote s.e. *p < 0.05, **p < 0.01, ***p < 0.001 for proportion significantly greater than 0.5. (*a*) Proportion of courtship events directed at the novel female in the direct novelty experiment. From left to right: trial in which intact females differed in eye colour (*n* = 58); trial in which either intact novel or directly familiar female was painted (*n* = 88); trial in which females were decapitated and pinned (*n* = 28). (*b*) Proportion of males mating with novel female first in the direct novelty experiment. Experiments are the same as in panel (*a*). From left to right, the total number of males that mated = 28, 40 and 6. There are no standard error bars for wild-type males with decapitated females as all of the males mated with the novel female (see Results) and therefore s.e. = 0. (*c*) Proportion of courtship events directed at the novel female in the phenotypic novelty experiment. Focal males were first mated to a female and subsequently presented with two females; one full-sibling of the first female who had developed in the same vial as that female (phenotypically familiar), and one female who was unrelated to the first female and had developed in a different vial (phenotypically novel). There was a significant difference in proportion of courtship between wild-type and *Orco*^1^ males for the ‘decapitated females’ experiment (denoted by horizontal line). From left to right, *n* = 36, 35, 79 and 70. (*d*) Proportion of males mating with the novel female first in the phenotypic novelty experiment. Legend is the same as that of panel (*c*). From left to right, the total number of males that mated = 36, 35 and 9. Datum for *Orco*^1^ males with decapitated females is not shown owing to the small sample size: only two matings occurred, both with the phenotypically familiar female. Values plotted in panels (*a*) and (*c*) are predicted values from the statistical models.

**Figure 2.**
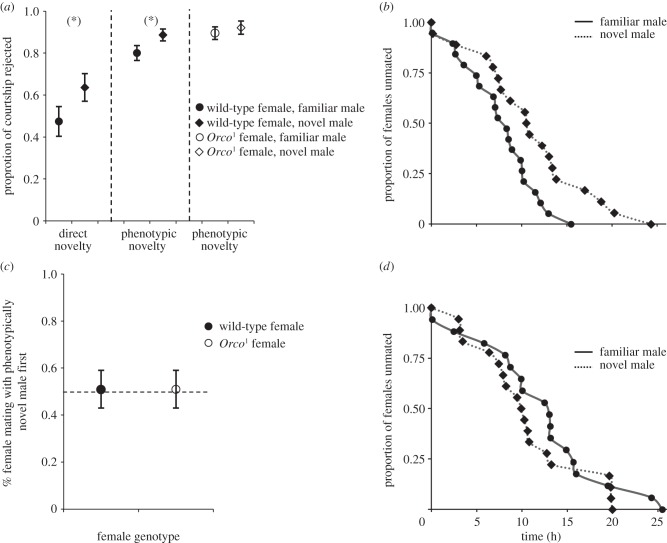
Female responses to direct and phenotypic familiarity of males. (*a*) Proportion of courtship rejected by females. From left to right: ‘direct familiarity’ experiment in which wild-type females were presented with a previous mate and novel mate (*n* = 43); ‘phenotypic novelty’ experiment in which wild-type females were presented with a full-sibling of their first mate who had developed in the same vial as that male (phenotypically familiar), and one male who was unrelated to the first mate and from a different vial (phenotypically novel; *n* = 59); ‘phenotypic novelty’ experiment in which *Orco*^1^ females were presented with a brother of their first mate who had developed in the same vial as that male (phenotypically familiar), and one male who was unrelated to their first mate and from a different vial (phenotypically novel; *n* = 56). Dashed lines separate different experiments. Asterisk (*) equals 0.05 < *p* < 0.10 for tests for difference in proportion of courtship rejected between familiar and novel males. *p*-values from left to right are 0.088, 0.082 and 0.176. (*b*) Cumulative survival functions for wild-type females. Likelihood ratio tests revealed a significant difference in remating latency with phenotypically familiar and phenotypically novel mates (*n* = 40, 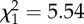, *p* = 0.019). (*c*) Proportion of females mating with a novel male first in the phenotypic novelty experiment. Focal females were either wild-type or *Orco*^1^ and were presented with a brother belonging to the same local environment as the first mate (phenotypically familiar) and one non-sibling belonging to a different local environment as the first mate. (*d*) Cumulative survival functions for *Orco*^1^ females. Likelihood ratio tests revealed no significant difference in remating latency with phenotypically familiar and novel mates (*n* = 35, 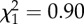, *p* = 0.344). Error bars denote s.e.

The *Orco* gene encodes for an olfactory co-receptor essential for olfaction [14,18,28]. Focal individual homozygous for *the Orco*^1^ mutation are therefore unable to use olfaction for discriminating between phenotypically familiar and phenotypically novel mates. To explore the potential role of olfactory senses in sex-specific response to phenotypic familiarity, we replicated the experiments on phenotypic familiarity with *Orco*^1^ focal individuals and recorded sex-specific response as outlined above (see the electronic supplementary material, figure S1*c*). Non-focal individuals were wild-type Dahomey. Sample sizes for each individual experiments are given in the legends of figures 1 and 2.

## 3. Results

### (a) Male response to direct novelty

When placed with two females—one recently mated by the focal male (directly familiar) and one recently mated to a different male (directly novel)—males preferentially courted the directly novel female (experiment 1: 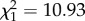, *p* < 0.001; experiment 2: 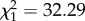, *p* = < 0.001; figure 1*a*). However, despite the bias in male courtship effort, males were not more likely to mate with directly novel females first (experiment 1: 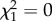, *p* = 1; experiment 2: 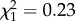, *p* = 0.631; figure 1*b*), suggesting that male courtship alone does not predict mating success, and that females may also play an important role. Males also courted white-eyed females significantly more than red-eyed females (experiment 1), and painted females more than non-painted females (experiment 2; see the electronic supplementary material, table S1).

When females were decapitated to control for their behaviour, we again found that males direct significantly more courtship to directly novel females (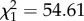, *p* < 0.001; figure 1*a*), confirming the male preference for directly novel females. Moreover, although few matings occurred in this experiment because decapitated females are deficient in their acceptance response [29], all of the forced matings were with directly novel females (directly novel = 6, directly familiar = 0; Fisher's exact test, *p* = 0.023; figure 1*b*). This suggests that only in the absence of female control, males are able to realize their preference for directly novel females and bias mating probability in favour of these females.

### (b) Male response to phenotypic novelty

When exposed to a female belonging to the same family and environment as the male's previous mate (i.e. phenotypically familiar) and a female belonging to a different family and environment from the male's previous mate (i.e. phenotypically novel), males directed more courtship towards the phenotypically novel female (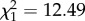, *p* < 0.001; figure 1*c*). As in the experiments investigating male responses to direct familiarity, we did not find that male preference for phenotypically novel females had an effect on the probability of first copulation (see the electronic supplementary material, table S2; figure 1*d*). We also found no effect of ‘marked status’ (paint colour) on the response variables measured and no significant interaction between ‘marked status’ and ‘familiarity’ in this experiment (see the electronic supplementary material, table S2). Experimental control of female behaviour, by decapitation and immobilization, confirmed the finding that males preferentially court the phenotypically novel of the two females (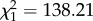, *p* < 0.001; see the electronic supplementary material, table S1; figure 1*c*). Matings with decapitated females were again rare: seven matings occurred with phenotypically familiar females and two with phenotypically novel females (Fisher's exact test, *p* = 0.167; figure 1*d*).

Taken together, these results show that males preferentially court directly and phenotypically novel females.

### (c) Female response to direct novelty

When examining female responses to direct and phenotypic familiarity, females presented with two males, one directly novel and one directly familiar, showed no preference for novel males. Instead, there was a marginally non-significant trend in the opposite direction for females to reject the courtship of the novel male more frequently than that of the familiar male (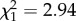, *p* = 0.087; figure 2*a*). This was not owing to a difference in courtship intensity between directly familiar and directly novel males (GLMM, 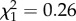, *p* = 0.610). There was no effect of ‘marked status’ (paint colour) on proportion of courtship rejected and no significant interaction of ‘marked status’ with ‘familiarity’ (see the electronic supplementary material, table S1).

### (d) Female response to phenotypic novelty

Female responses to phenotypic familiarity were also consistent with a lack of preference for novelty, and females again displayed a trend for neophobia: when exposed to two sexually novel virgin males, one phenotypically familiar and one phenotypically novel, females remated faster with the phenotypically familiar male than with the phenotypically novel male (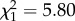, *p* = 0.016; figure 2*b*), and there was a marginally non-significant trend for females to reject a higher proportion of courtship by the phenotypically novel male than that by the phenotypically familiar male (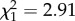, *p* = 0.088; figure 2*a*). Again, this was not owing to differences in courtship intensity between male types (former experiment, 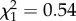, *p* = 0.461; latter experiment, 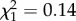, *p* = 0.709). Consistent with the results of male preference (see above), the probability of first mating did not differ between familiar and novel males (see the electronic supplementary material, table S2; figure 2*c*). We found no effect of ‘marked status’ (paint colour) on any of the response variables and no significant interaction with ‘familiarity’ (see the electronic supplementary material, tables S1 and S2).

### (e) Responses to phenotypic novelty in olfactory mutants

*Orco*^1^ mutant males performed significantly fewer courtship events than wild-type males in the experiment with decapitated females (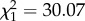, *p* < 0.001, mean ± s.e., wild-type: 12.59 ± 0.86, *Orco*^1^: 6.30 ± 0.51) but not in the experiment with intact females (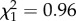, *p* = 0.327, mean ± s.e., wild-type: 2.94 ± 0.48, *Orco*^1^: 2.74 ± 0.53). This implies that olfactory cues can act as a sexual simulation for the males [30] and that olfaction is particularly important when the female is not moving and reacting, i.e. decapitated and immobilized.

We found that, in contrast to wild-type males, *Orco*^1^ mutant males did not bias courtship towards phenotypically novel females (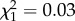, *p* = 0.873; figure 1*c*). As expected, the first female that males mated with was equally likely to be phenotypically familiar or phenotypically novel (see the electronic supplementary material, table S2; figure 1*d*). When exposed to decapitated females, *Orco*^1^ males courted the phenotypically novel female more frequently than the phenotypically familiar female (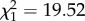, *p* < 0.001; see the electronic supplementary material, table S1; figure 1*c*), but this bias in courtship was significantly weaker than in wild-type males (preference in wild-type males versus preference in *Orco*^1^ males; 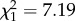, *p* = 0.007; figure 1*c*). Only two matings occurred with decapitated females in the experiments using *Orco*^1^ males, both of which were with phenotypically familiar females. Similarly, *Orco*^1^ females showed no sexual preferences in relation to phenotypic familiarity: there was no significant difference in rejection rate directed towards either phenotypically familiar or phenotypically novel males, (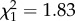, *p* = 0.176; figure 2*a*), no difference in the latency to remate with either male type (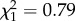, *p* = 0.373; figure 2*d*) and no difference in the probability of mating (see the electronic supplementary material, table S2; figure 2*c*). As with the experiments on wild-type females, we detected no difference in courtship intensity between male types (experiment on female rejection rate, *Orco*^1^: 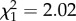, *p* = 0.155; experiment in vials, *Orco*^1^: 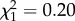, *p* = 0.655). There was also no effect of ‘marked status’ on the response variables measured and no significant interaction between ‘marked status’ and ‘familiarity’ in these experiments.

## 4. Discussion

### (a) Sex-specificity of responses to direct and phenotypic novelty

Our results demonstrate that both direct and phenotypic sexual familiarity play a key role in mate choice: males preferred to court directly and phenotypically novel females, and females displayed weak preferences for directly and phenotypically familiar males. Thus, in both sexes, the responses to direct familiarity and phenotypically familiarity of potential mates are in the same direction and further indicate that sexes differ in their response to direct and phenotypic familiarity of mates. Our results provide evidence for a male Coolidge effect in the fruit fly: an elevated interest by males in sexually novel over sexually familiar females [1]. This effect arises in many taxa [1,31], including another insect (burying beetle, *Nicrophorus vespilloides*; [32]).

The preference for phenotypically novel females observed in our fruit fly population might reflect a widespread male behaviour. For example, male sweat bees *Lasioglossum zephyrum*, when previously exposed to a female, elicit more mating attempts with a second female if she is less genetically related to the first female [29]. However, males in the sweat bee study were prevented from mating with the first female, so it is not clear whether the male preference is linked to mating. Male preferences for phenotypically novel females might be the consequence of avoidance of previous mates (e.g. as a potentially non-adaptive by-product of the Coolidge effect). Another, non-mutually exclusive explanation for a male preference for phenotypically novel females is that males might benefit by mating with dissimilar females through the higher genetic diversity of their offspring [33]. However, a recent study suggests that the benefits of phenotypically diverse offspring in the fruit fly may not be straightforward [34].

The functional significance of the weak female preferences for directly and phenotypically similar mates is also currently unclear. In fact, this result seemingly contrasts with recent studies which show that female fruit flies mate more frequently in groups composed of males from more than one laboratory strain [35] and discriminate against mating with socially familiarized males [36]. However, we do not know to what extent differences between laboratory strains, whose genetic differentiation is unknown (as in [35]), can be compared to differences between phenotypically novel and phenotypically familiar individuals within a population (as in our study). Similarly, we do not know to what extent non-copulatory experiences (as in [36]) can be compared to copulatory experiences (our study) in their impact upon future sexual behaviour. Female preferences for phenotypically similar mates in our study are also in contrast to predictions of the ‘rare male effect’, whereby males of a rare genotype attain higher mating success [37]. The rare male effect has been reported in many laboratory studies of *Drosophila* species [37,38], but may be less common than was actually supposed owing to problems with observer bias and lack of repeatability both with experimental design and with data analysis [37,38]. Our study suggests that females may actually prefer to mate with new males that are phenotypically similar to their previous mates, and that choice exerted by females might not account for the rare male effect.

Female preferences for phenotypically familiar males may be to avoid the potential costs of mating with phenotypically varied males. For example, in some species, females may incur costs from seminal diversity [39], or from mating with males that are unrelated to each other [40]. Another intriguing possibility is that female preference for phenotypically familiar mates might be the result of manipulation of female behaviour by the mating male (e.g. through seminal fluid peptides). This could potentially increase the chances that if the ejaculate of a focal male is to face sperm competition, such competition is restricted to males that are more related to the focal male than the average male in the population. These hypotheses should be explored in future studies.

Although we detected clear biases in male courtship and female latency to mating and rejection behaviour, measures of mating success and copulation duration in the ‘vial’ experiments did not vary between treatments (see the electronic supplementary material, table S2). Our results indicate that males and females have opposing preferences over direct familiarity of mates, and thus the lack of mating bias might be a result of these opposing preferences cancelling each other out. Therefore, assays of mating frequency might not reveal this potential hidden conflict between the sexes. Evidence for biases in mating success was only apparent in the experiment investigating direct familiarity with immobilized females. Owing to the rarity of copulations with decapitated females, we had low power for detecting biases in matings when female sexual behaviour was abolished. In our ‘phenotypic familiarity’ experiments, where males were presented with two potential mates, both female were virgins and therefore receptive to mating. Males could therefore mate with whichever female is willing to mate first, which is likely to be random when females are sexually naive. However, when we decapitated females, considering both direct and phenotypic novelty experiments together (figure 1*b*,*d*), male mating success was biased towards novel females (sum of matings with decapitated females across both direct and phenotypic novelty experiments: familiar = 2, novel = 13, Fisher's exact test, *p* = 0.007). Thus, mating success cannot be attributed exclusively to either male or female response [36]. Mating duration, though traditionally thought to be mainly under male control [41] can be modulated by female genotype [42], and thus would not reflect male-specific mating response. Therefore, courtship effort as an indication of male's preference, and remating latency as well as rejection propensity as measures of female's receptivity is probably a more precise representation of sex-specific biases in sexual behaviour [24,43,44] than measuring mating outcomes alone. It is conceivable that the observed sex-specific responses to the phenotypic familiarity of potential mates might influence mating success under natural conditions and/or in larger social groups, where individuals differ in their mating history and interact with multiple opposite-sex conspecifics. In addition, in wild populations, multiple males would be competing for the same female and it would be interesting to examine the effect of intra-sexual competition on male responses to direct and phenotypic familiarity. For example, a male might act more aggressively to a novel male competitor in the presence of the previous female mate [45]. Nevertheless, our study highlights the importance of exploring sexual behavioural mechanisms, rather than simply measuring outcomes such as mating success, in order to reveal biases in sexual behaviour and potential sexual conflict over mating.

### (b) The role of olfaction

Our results show that *Orco* is required for males and females to display full preferences for phenotypically novel or familiar members of the opposite sex. This suggests that sex-specific responses to phenotypic familiarity are at least in part controlled by olfactory cues, and indicates that olfaction may be needed for discrimination between individual potential mates. Olfactory signals are important mediators of species and sex discrimination among many insects [15,46], and as most insects rely on olfaction as the predominant sensory modality, their chemosensory systems have been fine-tuned to high levels of sensitivity and specificity [47]. Thus, insects have the potential for distinguishing individual differences in pheromonal make-up. In contrast to our knowledge of individual recognition in mammals [48,49], we still know little about the mechanism mediating individual discrimination in insects [50,51]. Our results lay the foundations for future work that can focus on establishing the potential role of specific pheromones in mate discrimination at the individual level in the fruit fly. The fruit fly also uses a variety of senses—vision, hearing, touch, taste and smell—to assess individuals and mediate sexual behaviours [52,53]. The small, but significant preference for phenotypically novel mates by *Orco*^1^ males in the beheaded female trials suggests that non-olfactory cues may also play a role in mediating this behaviour. Future studies should aim to elucidate the relative importance of multiple senses.

In our experiment, phenotypically familiar mates belonged to the same family and local environment as the previous mate and we did not partition the effects of genetic relatedness and common developmental environment on mating responses. Given that cuticular hydrocarbons are sensitive to environmental factors [54–56], two individuals collected from the same vial may have similar cuticular signatures even if they were genetically unrelated. This could be a result of individuals from a particular vial (at densities of 20–30 individuals per vial) frequently rubbing against one other and exchanging cuticular hydrocarbon signatures, which might also reflect the scenario in the wild where larvae aggregate on rotting fruit. It will be particularly intriguing to disentangle the effects of genetic familiarity and environmental factors on mating preferences in future studies.

## 5. Conclusion

Our results show that male and female *D. melanogaster* respond behaviourally, and in opposing directions, to the direct and phenotypic familiarity of their potential mates. Behavioural responses to the direct and phenotypic familiarity of potential mates are likely to evolve in species with limited or sex-biased dispersal prior to mating because this increases the probability of interacting with a previous mate or their previous mate's relatives. It will be important to determine to what extent these type of responses are shared, or differ, in other taxa, and how this relates to patterns of dispersal and interaction rates. Our findings show that male and female fruit flies have divergent responses to direct and phenotypic familiarity, indicating that selection may have acted differently on the sexes. Intriguingly, sex-specific differences in sexual behaviour did not translate into difference in mating success except when the behaviour of one sex was experimentally inhibited. Further research is therefore needed to determine whether there are significant fitness consequences of these behaviours and to uncover the underlying evolutionary dynamics. Finally, our data show that both sexes may use olfaction in choosing which individuals to sexually pursue or resist. This opens the door to elucidation of the specific mechanisms underlying familiarity recognition in this key genetic model organism.

## Data accessibility

All data are available on dryad.
